# Automatic Changes Detection between Outdated Building Maps and New VHR Images Based on Pre-Trained Fully Convolutional Feature Maps

**DOI:** 10.3390/s20195538

**Published:** 2020-09-27

**Authors:** Yunsheng Zhang, Yaochen Zhu, Haifeng Li, Siyang Chen, Jian Peng, Ling Zhao

**Affiliations:** 1 School of Geoscience and Info-Physics, Central South University, Changsha 410083, China; zhangys@csu.edu.cn (Y.Z.); lehaifeng@csu.edu.cn (H.L.); siyangchen@csu.edu.cn (S.C.); PengJ2017@csu.edu.cn (J.P.); 2 School of Remote Sensing and Information Engineering, Wuhan University, Wuhan 430072, China; 0107150110@csu.edu.cn

**Keywords:** changes detection, fully convolutional feature maps, outdated building map, VHR images

## Abstract

Detecting changes between the existing building basemaps and newly acquired high spatial resolution remotely sensed (HRS) images is a time-consuming task. This is mainly because of the data labeling and poor performance of hand-crafted features. In this paper, for efficient feature extraction, we propose a fully convolutional feature extractor that is reconstructed from the deep convolutional neural network (DCNN) and pre-trained on the Pascal VOC dataset. Our proposed method extract pixel-wise features, and choose salient features based on a random forest (RF) algorithm using the existing basemaps. A data cleaning method through cross-validation and label-uncertainty estimation is also proposed to select potential correct labels and use them for training an RF classifier to extract the building from new HRS images. The pixel-wise initial classification results are refined based on a superpixel-based graph cuts algorithm and compared to the existing building basemaps to obtain the change map. Experiments with two simulated and three real datasets confirm the effectiveness of our proposed method and indicate high accuracy and low false alarm rate.

## 1. Introduction

Developing countries have witnessed a rapid expansion of urban areas during the last decades. With the fast urbanization, updating buildings geo-database plays an important role in urban planning, as it provides valuable information regarding, e.g., land use/cover monitoring [1], evaluation of agricultural lands decline [2], disaster assessment [3], civil BIM updating [4]. Such information also enables the government to adopt suitable and sustainable development strategies. Automatic building geo-database updating relies on identifying the areas, where changes occurred. Currently, change identification is mainly a labor-intensive work, especially in urban environments, due to its complexity. Therefore, automatic geo-database updating based on remote sensing images remains an open and unsolved issue.

During the past decades, several methods have been proposed to increase the level of automation in change detection. According to their comparison basis, the change detection methods can be categorized into two classes: (1) Image-image comparison; and (2) image-map comparison [5]. The former approach aims at direct recognition of differences between multi-temporal remotely sensed images [6,7]. The image-map comparison-based method, however, detects changes between existing data and newly acquired images, where the semantic classification of the newly acquired images is also required. For image-map comparison, supervised machine learning methods are employed, see, e.g., Reference [8]. However, for an accurate classifier to be trained, a large enough set of labeled samples is required. Labeling samples, however, need expensive manual work and a high level of expertise and knowledge on image interpretation.

To address this issue, existing GIS data or online maps, such as Open Street Map (OSM) data, and Google maps, are employed to provide prior information. For example, Bouziani et al. obtain prior class knowledge from the existing geo-database to identify the change of buildings based on transitional probability between classes, and to change map segmentation [5]. Kaiser et al. exploit the online map to guide aerial image segmentation, although they simply ignore the temporal inconsistencies between the used map and aerial images, and simply count on human interaction to remove the mis-registrations between the map and the roof images of buildings [9]. Wan et al. employ OSM data to obtain initial samples for training SVM to classify HRS images [10]. To alleviate the effect of intrinsic errors caused by incorrect labeling by volunteers, they further use a cluster analysis to filter out the possible errors. Gevaert et al. provide a model for outdated base-maps as noisy labels of newly acquired UAV images, and then utilize data cleansing methods to filter out the potentially mislabeled samples, and further re-predict their labels by supervised classification [11]. Chen et al. treat historical digital line graph (DLG) data as the source of initial noisy labels, and then the pure part is selected by an iterative training method [12]. For highly accurate classification, they also use several hand-crafted image-based and point-cloud based features for the supervised classification task. The elevation feature is also very useful to distinguish buildings; however, it is not always available.

In addition to the availability of a large enough set of labeled samples, selecting proper discriminable features is another key point for classification. Some carefully hand-crafted features are heuristically proposed and combined to classify VHR images. Most of the existing methods employ spectral and textural features, or DEM data, as feature descriptors, see References [11,13,14]. Although the hand-crafted features are designed to describe a specific image pattern, their performance depends on the available training data. Different from hand-crafted features, the recently developed deep learning techniques directly learn features from the original data. Deep learning is widely used in various research areas, e.g., natural image classification [15], object detection [16], and semantic segmentation [17]. Deep learning methods are also used to learn features from remote sensing (RS) images for classification [18]. For instance, autoencoder-based techniques are used in RS for extracting features from images [19,20,21]. Such methods learn to extract feature encodings in an unsupervised setting, which can then be reconstructed back to the input with minimum error [21]. Different variations of autoencoders are applied to various tasks in the RS field. By increasing the spatial resolution of the RS images, the training of such autoencoders becomes time-consuming and further requires large memory.

In practice, a large set of accurately labeled data is often unavailable. In recent works, this issue is addressed in the RS domain by training deep convolutional neural networks (DCNNs) from scratch. Feature extraction DCNNs is also widely used in computer vision research, where the training is based on large open-source datasets, see References [22,23]. The intuition behind DCNNs is that with strong learning abilities, DCNNs can learn to respond to various kinds of feature patterns in different abstract-levels from large and complex datasets. The learned features can then be generalized to be used for smaller datasets, even if those datasets are remarkably different from the training datasets [24]. Much research has been done to generate a single feature descriptor for the whole image with high-level activations of pre-trained DCNNs [25]. In these methods, the size of the input is strictly fixed, so interpolations are needed to resize the images to a specified scale. To extract dense feature maps in a pixel-wise fashion, such methods need to crop window, resize, and do forward propagation at the center of each pixel [20,26]. Since most of the computation in the neighboring windows are shared through the convolution, they are computationally redundant and limited to small/moderate-size images. Many existing methods focus on extracting features from the back part of DCNNs (i.e., the last convolutional layer and fc layers) and generate one single feature description for the whole image.

To improve classification performance, the spatial context of the images has to be fully used [23,27]. Single-pixel based methods are unable to take a large enough image field to distinguish the building objects from the background information and ensure a consistent classification result in the global context. Several pixel-based methods are proved to be successful for change detection of low- and moderate-resolution remotely sensed images [7]. Nevertheless, with the emergence of high-resolution remote sensing (HRS) data, such methods are not effective, since the results can easily keep salt-and-pepper noise, due to increasing (decreasing) intra-(inter-)class variance [28]. To address this issue, object-based methods are adopted in References [29,30,31,32]. Such object-based change detection methods significantly reduce the required amount of data to be processed, and further generate change recognition result with shape and boundary information that can be directly used to update geo-databases, see Reference [33]. This however may lead to new problems as object segmentation is intrinsically challenging for remote sensing images [34].

In this paper, we propose to cast the image-map change detection problem into the identification and correction of noisy labels. For extracting discriminable features, a fully convolutional network (FCN) pre-trained on the PASCAL VOC dataset [17] is treated as a fully convolutional feature extractor (FCFE). Since the long-range relationship comparatively is trivial in the HRS images, and spatial information is severely lost by down-sampling in the last convolutional layers, only first two groups of convolutional layers (4 layers) are preserved. The tensors from all convolutional layers are then up-sampled to the same size of the input and fused together by concatenation as pixel-wise features. Through FCFE, the feature computation of all pixels is achieved by a single forward propagation. Therefore, it is more efficient than that of the most window-based feature extractors. However, directly concatenated and up-sampled pixel-wise features are redundant and have a high dimension for subsequent processing. Therefore, a noise label guided feature selection is proposed to select the most informative features for building extraction. As pixel-wise re-predicted labels of newly acquired HRS images are usually fragmented, especially in areas with a similar spectral, textural characteristic, such as buildings, roads, and bare soil. To alleviate this problem, new HRS images are segmented into superpixels, and then superpixel-based graph cuts are used to refine the initial classification result. For further performance improvement, we also propose a new label uncertainty calculation technique for each superpixel.

The contribution of our work are the following: (1) We present a novel framework with the combination of pixel-wise and object-based analysis for image-map change detection based on data cleaning method; (2) FCN pre-trained on the PASCAL VOC dataset for semantic segmentation is then used to reconstruct the proposed fully convolutional feature extractors to extract dense features of HRS images; and (3) outdated noise label is then used to guide the feature selection for eliminating the redundancy of the features.

The remainder of this paper is organized as the following. Section 2 provides the details of the proposed image-map change detection framework. Section 3 analyses the performance of experiments conducted on two simulated, and three real datasets. Finally, conclusions are presented in Section 4.

## 2. Methods

### 2.1. Overview of the Method

**Figure 1:**
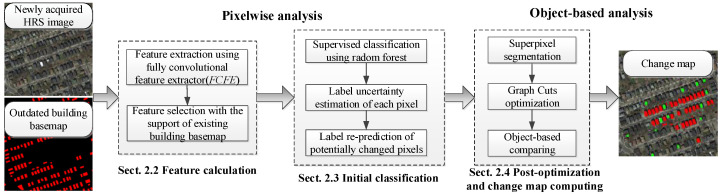
Flowchart of the proposed change detection framework. HRS, resolution remotely sensed.

The workflow of the proposed approach is illustrated in Figure 1, where the three main components are:

(1) Feature calculation, which is a fully convolutional feature extractor reconstructed from FCN-8s [17] and pre-trained on the PASCAL VOC dataset. Feature calculation extracts multi-scale pixel-wise features from newly acquired HRS images. An RF classifier is then trained to rank the importance of the extracted features based on the outdated basemap. After that, representative features are selected as feature descriptors for each pixel.
(2) Initial classification, where the label uncertainty for each pixel is estimated through cross-validation based on selected features. The reliable (unchanged) pixels are then separated as training samples to train the new RF classifier, and potentially changed pixels are re-predicted.
(3) Post optimization and change map computing, where the SLIC (Simple Linear Iterative Cluster) algorithm [35] is used to segment HRS images into superpixels, and the probability of superpixels for each label is estimated. The negative logarithm of probability is then used to construct the data term. A Gaussian kernel of normalized RGB feature is then used to construct a smooth term of the energy function. After that, the graph cuts algorithm is used to minimize the energy function and obtain the optimized, updated label. The updated labels are finally compared with the outdated basemap to compute the change map.

### 2.2. Feature Extraction through Fully Convolutional Feature Extractor

Although the last layers of CNNs are more effective in capturing semantics, they are ineffective in capturing fine-grained spatial details, which are needed for spatial feature extraction [36]. Two obstacles that hinder the direct transformation of DCNNs into dense feature extractors are: (1) Pooling layers shrink features maps exponentially, and this depresses valuable spatial information; (2) fc layers map fix-size feature tensors into activation vectors, this constrains the input size. In computer vision, images are relatively small and contain only a few salient objects and/or one main scene. This makes cascaded down-sampling important to extract relationships within the main objects. However, HRS images contain objects this belong to different categories, and there exists no single subject being able to globally determine the theme of HRS images. Therefore, long-range relationships captured by stacked pooling layers seem trivial, but the local response captured by the early convolutional layers (convlayer) is much more important.

Convolutional kernels in DCNNs pre-trained on a very-large dataset are considerably rich filter banks capturing various kinds of features. Zeiler and Fergus demonstrate that the early convlayer encodes low-level features, such as edges, corners, shapes, or textures, while the deeper layers extract high-level information, such as objects, or categories [37]. Kemker et al. assert that the features extracted by the convlayer of the pre-trained DCNNs can produce Gabor-like results [38]. Generally, feature maps extracted by the deeper convlayer are coarse and abstract, suffer from a severe size reduction, and contain more information of the source datasets, which is irrelevant when transferring to a new target dataset. Nevertheless, feature maps extracted from the earlier layers are fine-grained and adhere better to the boundaries. Therefore, one can assume that the features from early convlayers of pre-trained DCNNs have stronger generalization abilities [39]. Since convlayers also accepts arbitrary input size and intrinsically preserves spatial information, fully convolutional networks (FCN) reconstructed by the early part of pre-trained DCNNs are more efficient to extract dense features.

**Figure 2:**
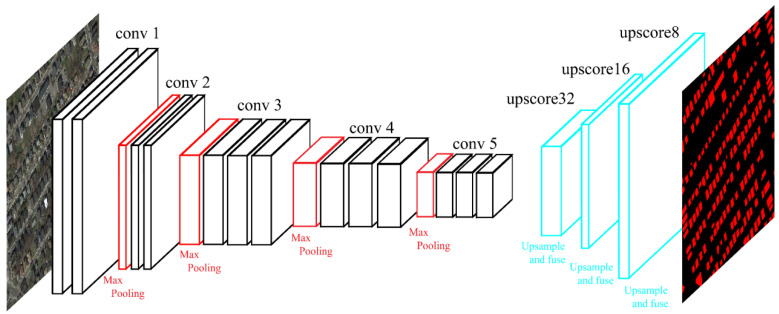
Structure of the original fully convolutional network (FCN)-8s [17].

FCN-8s [17] is an FCN pre-trained on the PASCAL VOC dataset for 20-class semantic segmentation, is used to reconstruct the proposed fully convolutional feature extractors (FCFE). The used FCN-8s is trained on the PASCAL VOC 2011 segmentation challenge training set, which includes 11,530 images and 5034 segmentations. It is reconstructed and fine-tuned from VGGNet [40] that is pre-trained on ImageNet. FCN-8s consists of five groups of convlayers with pooling layers that encode the input image into high-dimensional dense feature maps. It also has three deconvolutional layers that up-sample and fuse activations from the last three pooling layers to the size of the input as the predictions. The structure of the original FCN-8s is illustrated in Figure 2.

#### 2.2.1. Structure of the Proposed Fully Convolutional Feature Extractor

**Figure 3:**
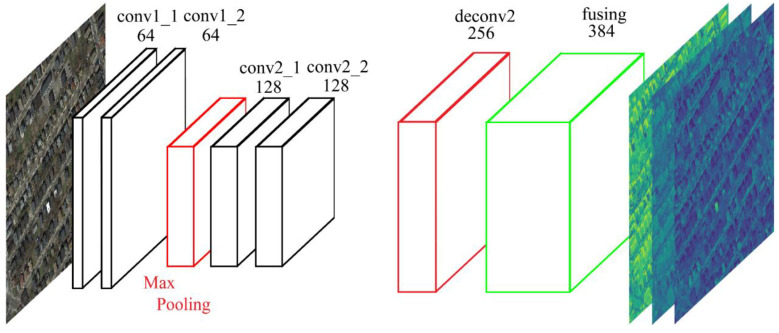
Structure of the proposed FCFE.

The structure of the proposed fully convolutional feature extractor is illustrated in Figure 3. To reconstruct pre-trained FCN-8s for dense feature extraction tasks, we make the following three modifications: (1) The feature maps extracted by convlayers after the pool2 layer are coarse (i.e., one-sixteenth the size of original image), and assumed to contain more information about source dataset. Therefore, only the first two groups of convlayers with the first pooling layers are preserved. This modification is aimed to exploit multi-level well-generalized features, while preserving valuable spatial information. (2) In the original FCN-8s, the first convlayer zero-pads the input image with 100 pixels to prevent severe size-reduction imposed by cascaded pooling layers. Other convlayers also pad the input feature map with 1 pixel. Note that all convolution kernels in FCN-8s are 3 × 3 in size, and their output has exactly the same spatial dimension as the input. In our fully-convolutional feature extractor (FCFE), all convlayers are set to pad input the feature map with 1 pixel. Therefore, feature maps from the first group of convlayers have the same size as the input image, while feature maps from the last convlayers are two-times downsampled. (3) The feature map extracted from the last group of convlayers is upsampled to the input size using bilinear interpolation. All feature maps are then concatenated to multi-scale deep features.

In Figure 3, the multi-scale features extracted by FCFE are up-sampled and fused feature maps from conv1_1, conv1_2, conv2_1, and conv2_2 layers of PASCAL VOC dataset-pretrained FCN-8s model, with 64, 64, 128, and 128 channels, respectively. Layer deconv2 uses bilinear interpolation to upsample feature maps from conv2_1 and conv2_2 to the size of the input image and fuse them together. The fusing1 layer concatenates the feature maps from conv1_1, conv1_2, and deconv2 to obtain the final 384-dimensional multi-scale features.

#### 2.2.2. Feature Selection Guided by the Existing Basemaps Using Random Forest

Only part of the features directly extracted by the FCFE is highly discriminative for buildings, and the rest are redundant and high-dimensional. Therefore, direct feeding of the features into the subsequent data cleaning pipeline demands excessive computation, and also harms the data cleaning effects. According to the study in Reference [41], each feature layer generated by DCNN responds to a major class. Thus, the feature selection processing is performed to select the most informative features and ensure the classification result. Feature selection is the process of removing redundant and irrelevant features, often accomplished by determining the usefulness of all feature variables [42]. Feature selection methods can be generally classified into three categories, including supervised, semi-supervised, and unsupervised methods. The existing building basemaps may contain erroneously labeled areas, due to time-lapse with the newly acquired HRS image, however, the majority of the labels remain correct and can be used in the feature selection schemes.

Here we employ RF classifiers to select features in our proposed method. RF classifier trains multiple decision trees with a random subset of samples based on a random subset of features [43,44]. RF algorithm can be trained efficiently to process the multiple label classification problems, and it is widely used in RS image classification tasks [43]. RF also provides the importance of the used features. Therefore, the feature importance estimated by RF is the average importance of each decision tree.

In order to select the salient feature that discriminates well from the building to background pixels, 384-dimensional FCFE extracted features and existing building basemaps, as pixel-wise labels, are considered as the training set to fit an RF classifier. The features’ importance is then evaluated, and nch (experimentally set to be 20) most important features are selected chosen to form the feature descriptor of the newly acquired HRS image.

**Figure 4:**
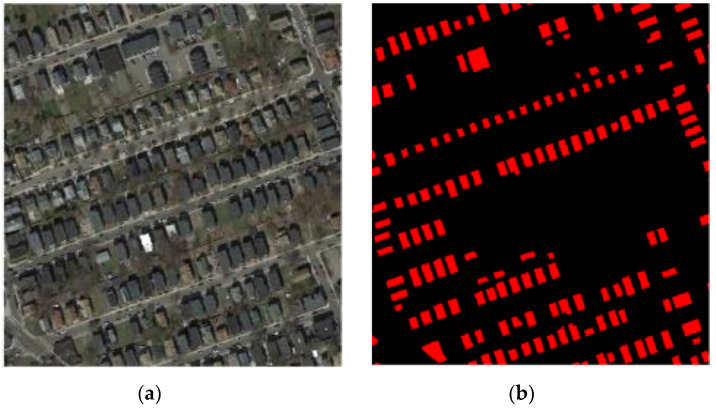
Example data for illustration of the proposed feature extraction and selection techniques. (**a**) Example image, and (**b**) outdated map.

**Figure 5:**
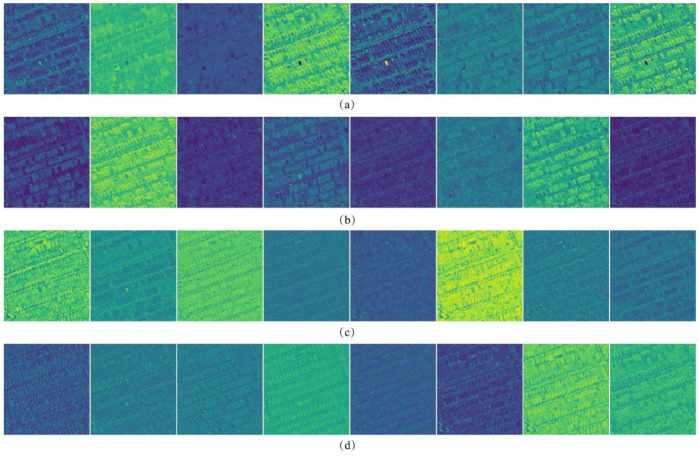
Eight randomly selected feature maps from each layer of the FCFE; (**a**) conv1_1; (**b**) conv1_2; (**c**) conv2_1; (**d**) conv2_2.

To visually analyze the features extracted by the proposed method, an image, as shown in Figure 4, is used to perform the FCFE and feature selection processing. To display and compare features inner-layer- and cross-layer-wise, eight features are randomly chosen from each layer, and a total number of 32 feature maps are illustrated in Figure 5.

By carefully examining Figure 5, three characteristics of the feature extracted by FCFE can be concluded: (1) A small part of the features is highly discriminative between buildings and background, with the corresponding feature maps showing salient contrast between the two classes; (2) a large number of features are less useful; with feature maps being ambiguous and showing inconspicuous differences; (3) features from early convlayers are fine-grained and adhere better to the boundaries, whereas features from latter convlayers are comparatively coarse and more abstract.

**Figure 6:**
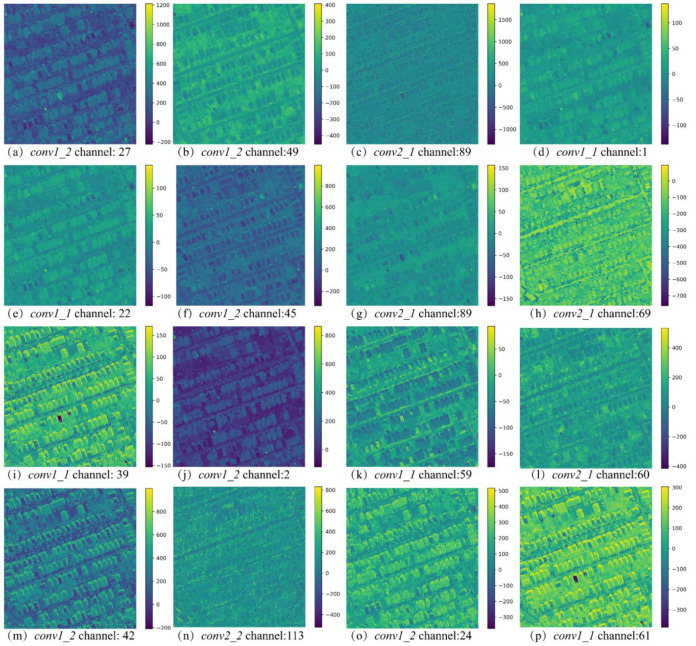
Sixteen most important features were selected by RF with the support of existing building basemaps.

Sixteen most important features chosen after feature selection are shown in Figure 6. Three properties of selected features can be seen in Figure 6: (1) By filtering the ineffective features out, the remaining features are more representative and visually separable; (2) selected feature maps are functionally versatile. It is also seen that (a,d,e,h,o) positively respond to the buildings, whereas (b,c,f,j,k) negatively respond to the buildings; and (I,m,p) strongly respond to shadows and are actually shadow detectors. Since the buildings are supposed to be near, where the shadows appear, the detection of shadows can positively support the recognition of buildings. (3) Features from four convlayers are all selected to form the multi-scale features. As stated before, features from early layers contain low-level knowledge, such as positions and boundaries, while features from latter layers encode high-level intuitions, such as neighboring and contextual information. Based on that, the selected features are complementary and representative, and they are combined into a feature descriptor for HRS images.

### 2.3. Initial Classification by Automatic Sample Selection Using RF

As noise label is used to guide the feature selection. This however may harm the classification result compared to the pure label. Therefore, the existing basemaps are viewed as noisy labels of newly acquired HRS image; then, the selected deep features are utilized to purify the initial labels through a data cleaning procedure.

In the field of machine learning, data cleaning is often introduced in the classification task with noisy labels, and intends to identify and correct mislabeled samples [45]. The core of the data cleaning idea lies in estimating the label uncertainty of each sample. Note that in the label uncertainty estimation step, the training data is also noisy. Therefore, classifiers that are robust to label noise are preferable. Most classifiers are highly sensitive to the label noise, such as SVM and AdaBoost. However, some algorithms can avoid the effect of label noise to an extent. As mentioned before, the random forest is an ensemble decision tree classifier that introduces randomness in both samples and features selection, which makes it more robust, thus suitable for data cleaning tasks.

Inspired by the work in Reference [46], we use a cross-validation algorithm to estimate the uncertainty of the samples’ labels. The pseudocode for estimating the uncertainty of the initial labels is given in Algorithm 1.
**Algorithm 1.** Label uncertainty estimation**Input**: *S* (sample set, i.e., pixel index from HRS image) with *F* (features from Section 2.2), *L* (noisy label acquired from the existing basemaps); *k_max_* (pre-defined times of dataset partition); *N_est_* (number of RF meta-estimators); *D_max_* (max depth of the decision trees in RF) **Procedure:**(1)Divide *S* into *S_pos_*, and *S_neg_* according to *L*.(2)Initialize *M_u_* as *N*-dimensional zero vectors as the label uncertainty estimator, N is sample capacity.
**For** k in range(*k_max_*):(3)Randomly divide *S_pos_* into equally-sized S*^k^_pos1_* and *S^k^_pos2_*. Almost equally-sized S*^k^_neg_* are randomly chosen from *S_neg_*.(4)Train RF classifier, *RF^k^_pos1neg_*, with *S^k^_pos1_* and *S^k^_neg_*. Predict the label of *S^k^_pos2_*, *C^k^_pos2_*. Update *M_u_* for negative *C^k^_pos_*(5)Estimate the label uncertainty of *S^k^_pos1_* that is similar to step (4).(6)Estimate the label uncertainty of *S_neg_* as (4), (5).
**End for** **Output:** Accumulator *M_u_* indicating the label uncertainty of *S*.

For supervised machine learning, equally-sized training samples for each class are preferable. However, in satellite images, the background usually occupies more space than that of the buildings. In order to adjust the bias introduced by unbalancing distribution of samples, a larger penalty is imposed on inconsistent label prediction results of the background samples, i.e.,

(1) $$M_{u}[L(S)\neq L_{p}(S)]=\{\begin{array}{cc} 1 & \mathrm{if}\;L(S)=\mathrm{pos}\\ \sqrt{N_{neg}/N_{pos}} & \mathrm{otherwise} \end{array},$$

where *M_u_* is an accumulative matrix describing label uncertainty of each sample, *L*(*S*) is the noisy label of S, *L_p_*(*S*) is the label predicted by the classifier, *N_neg_*, and *N_pos_* are the number of background, and building pixels, respectively.

After obtaining Mu, r=Muk is calculated for each pixel, then a pixel with *r* > 0.5 is a possible mislabeled sample. Otherwise, it is considered as a clean sample. Finally, these cleaned samples are used to train an RF classifier, rFfinal, to predict the label of potentially changed samples to building or other class. The label probability of each sample is also obtained by Rffinal, which is then used for subsequent post-processing.

### 2.4. Post-Optimization Using Graph Cuts and Change Map Computing

Since the data cleaning processing is conducted pixel-wise, and little contextual information is taken into account, the initial classification result is fragmented. To ensure neighborhood consistency, post-optimization processing is formulated as an energy minimization problem, and graph cuts [47] algorithm that are performed on superpixels instead of entire pixels are used to find the solution and ensure the efficiency.

Here we use the SLIC algorithm to segment the HRS image into superpixels. It is shown that SLIC generates compact superpixels adhering tightly to the boundary [35]. The probability of the superpixel belonging to each class (building or other) is then calculated using Equation (2). It includes two aspects: (1) The averaged label probability of pixels in the superpixel; and (2) the proportion of pixels belongs to the current class.

(2) $$p(L(Spix)=c)=0.5\times (\sum_{pix\in Spix}p(L(pix)=c)+\frac{\left|pix\in Spix,L(pix)=c\right|}{\left|pix\in Spix\right|})$$

where *Spix* is the superpixel, *pix* are the pixels belonging to *Spix*, *c* is the label of two defined classes, *L*(*x*) returns the label of x, and |*s*| is the number of elements in set s.

The basic idea of graph cuts is to incorporate prior knowledge of label assignment, and the penalty imposed on adjacent superpixels with different labels, into a weighted graph. We then construct an energy function on the graph, and the optimal label assignment is obtained by optimizing the energy function defined as:

(3) $$E=\sum_{i}D(c_{i})+\lambda \sum_{i<j}S(c_{i},c_{j}).$$

The first term, *D*(*c_i_*), is the data term which is determined by the negative logarithm of the probability obtained from Equation (3) and defined as

(4) $$D(c_{i})=-\log(p(L(Spix_{i})=c_{i}))$$

The second term in Equation (3), *S*(*c_i_*, *c_j_*), is the smooth term, imposing a penalty on adjacent superpixels with different labels according to their similarity. Metric of spectral difference, i.e., Gaussian kernel of the averaged RGB feature, is utilized as the similarity measurement. Since the longer boundary is shared between the two superpixels, the higher their influence will be on each other, the penalty is weighted on the mutual border length. The smooth term employed in this paper is defined as:

(5) $$S(c_{i},c_{j})=w(i,j)\times \exp\frac{\left(\left\|f_{i}-f_{j}\right\|\right)}{\sigma^{2}}\times \delta (i,j),$$

where

(6) $$w(i,j)=\frac{bon(i,j)\times \left|N(i)\right|}{\sum_{j\in N(i)}bon(i,j)},$$

(7) $$\delta (i,j)=\{\begin{array}{ll} 1 & \mathrm{if}\;c_{i}\neq c_{j}\\ 0 & \mathrm{otherwise} \end{array},$$

σ is the standard deviation of Gaussian Kernel; $f_{i}$, $f_{j}$ are the averaged RGB feature of *i*th and *j*th superpixels, respectively; bon(i,j) is the shared border length of the *i*th and *j*th superpixels; |N(i)| is the number of neighbors of superpixel *i*; and $c_{i}$ is the label of superpixel *i*.

The parameter, λ, in Equation (3) controls the proportion of smooth term in the energy function. The larger the value of λ, the heavier will be the penalty imposed on the adjacent superpixels with different labels. This leads to more smoothing effects. The value of λ is related to the size of buildings in HRS image. If most buildings are small, consisting of only a few superpixels, λ needs to be reduced to avoid over-smoothing of the building superpixels by the surrounding background superpixels. Otherwise, λ, is set to a larger value to introduce a better smoothing effect.

After building the energy function, the maximum flow of the graph [48] is obtained to get the minimum cuts and obtain the optimal label for each superpixel. After obtaining the final classification result of the new HRS images, the labels of the images are compared to the existing map to obtain the change map.

## 3. Experimental Results and Discussion

The proposed framework is implemented using python language. Pre-trained model weights of FCN-8s are obtained from (https://github.com/shelhamer/fcn.berkeleyvision.org) under caffe [49] framework and then transformed into tensorflow (https://www.tensorflow.org/) readable form, and reconstructed into fully convolutional feature extractor (FCFE). Graph cuts are implemented using PyMaxflow (https://github.com/pmneila/PyMaxflow).

### 3.1. Experiment Setup

#### 3.1.1. Datasets Description

**Figure 7:**
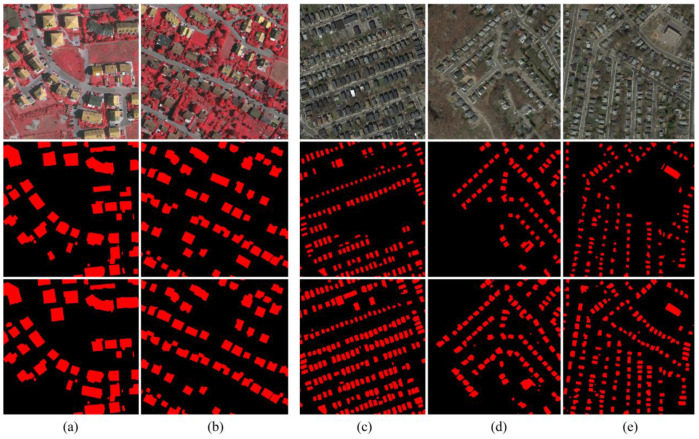
Experimental data sets: (**a**,**b**) ISPRS simulated dataset, (**c**–**e**) Boston real dataset (the first row is the newly acquired HRS image, the middle row is the outdated building map, and the third row is the ground truth building map for new HRS images).

To evaluate the proposed method, we use five datasets as shown in Figure 7, they include two sets, including ISPRS simulated dataset, and Boston real dataset—for details, see Table 1:

ISPRS simulated dataset: Two airborne images from ISPRS 2D semantic segmentation benchmarks (downloaded from http://www2.isprs.org/commissions/comm3/wg4/2d-sem-label-vaihingen.html) are employed to simulate two synthetic datasets as newly acquired HRS images. Approximately 10% of new building labels are randomly added. To simulate the outdated basemaps, 15% of the existing labels are deleted from the ground truth.

Boston real dataset: Three real datasets are selected from the urban areas of Boston, USA. The outdated basemaps are obtained from an existing classification dataset [50] (downloaded from http://www.cs.utoronto.ca/~vmnih/data/), and regions that contain obvious changes are cropped. Then the corresponding newly acquired HRS images are downloaded from Google Earth. The main challenges with this dataset are: (1) Backgrounds are heterogeneous and share spectral similarity with the buildings; therefore, pure pixel-based change detection may result in a high false-positive rate. (2) Buildings are relatively small; therefore, object-based strategies may suffer from instability of random classifiers. This may lead to false-negative outcomes. (3) Labels of the existing buildings suffer from severe mis-registration error, which makes information about building samples inaccurate. In order to evaluate the effectiveness of the proposed framework, an expert person is also invited to delineate the buildings’ boundaries from the HRS images. The results are then reviewed by another expert, both independent of the experiment.

#### 3.1.2. Assessment Criteria

In image-image change detection, the recognition result is a change map indicating the location of pixels that are notably different between multiple images. The result of image-map comparison is the updated label map. Similar criteria can be used to assess the accuracy assessment in both change detection techniques. In this paper, three evaluating indexes are obtained in pixel-wise fashion to evaluate the accuracy of the change detection result, including, completeness (Comp), false detection rate (FDR), and overall accuracy (OA):

(8) $$Completeness=\frac{C_{d}}{C_{t}},$$

(9) $$FDR=1-\frac{C_{d}}{C_{a}},$$

(10) $$OA=\frac{C_{d}+C_{n}}{C},$$

where $C_{d}$ is the number of changed pixels (both background to building and building to background) that are correctly detected, $C_{t}$ is the number of really changed pixels between newly acquired HRS image and the outdated basemap, $C_{a}$ is the number of all the pixels that are labeled differently in the new labeled map, and the outdated basemap, $C_{n}$ is the number of unchanged pixels that are correctly detected, and *C* is the number of pixels in the HRS image. Completeness measures the percentage of successfully corrected changed pixels among all changed pixels, whereas *FDR* reflects the proportion of false change pixels that are labeled as changed by the proposed algorithm. The *OA* also determines the comprehensive detection capability by taking both changed and unchanged pixels into account.

#### 3.1.3. Parameters Setting

There are three parameters having a high impact on the results. All these parameters are set based on trial and error. Unless otherwise stated, these parameters are used in our experiments.

The first one is a max depth of the RF classifier, *D_max_*, which determines the degree to which RF fits the training set. For a small *D_max_*, RF is under-fit to the training set resulting in a high variance. If *D_max_* is set to a large value, RF tends to over-fit to the mislabeled data in the training sets, resulting in a high bias. To balance the completeness and FDR, we set *D_max_* = 11.

Compared to *D_max_*, a number of decision tree estimators, *N_est_*, in RF has trivial effects on the data cleansing accuracy. For *N_est_* < 5, OA and FDR slightly fluctuate, due to the intrinsic randomness of the meta-classifiers, whereas for *N_est_* > 5, OA and FDR converge to a fixed level. Since the computational demands are linearly proportional to *N_est_*, we set its value to the minimum stable value of 5.

The main parameters of the post-optimization are the proportion of smooth term, λ, and the standard deviation of Gaussian kernel, σ.

Parameter λ controls the smoothness of the classification result. For a small λ, graph cuts tend to undersmooth the label results, and thus, holes and gaps of building labels and spurious fragmentations are under smoothened, causing a low completeness and OA, and a high FDR. For a very large λ, the label results are over smoothened and lots of existing buildings are obliterated, causing the bounce of FDR and re-sink of completeness and OA. Here, we set λ equal to 1.0 for ISPRS datasets, and 0.3 for Boston datasets. The value of σ is also set to 10.

### 3.2. Results of ISPRS Simulated Data

#### 3.2.1. Change Detection Results

**Figure 8:**
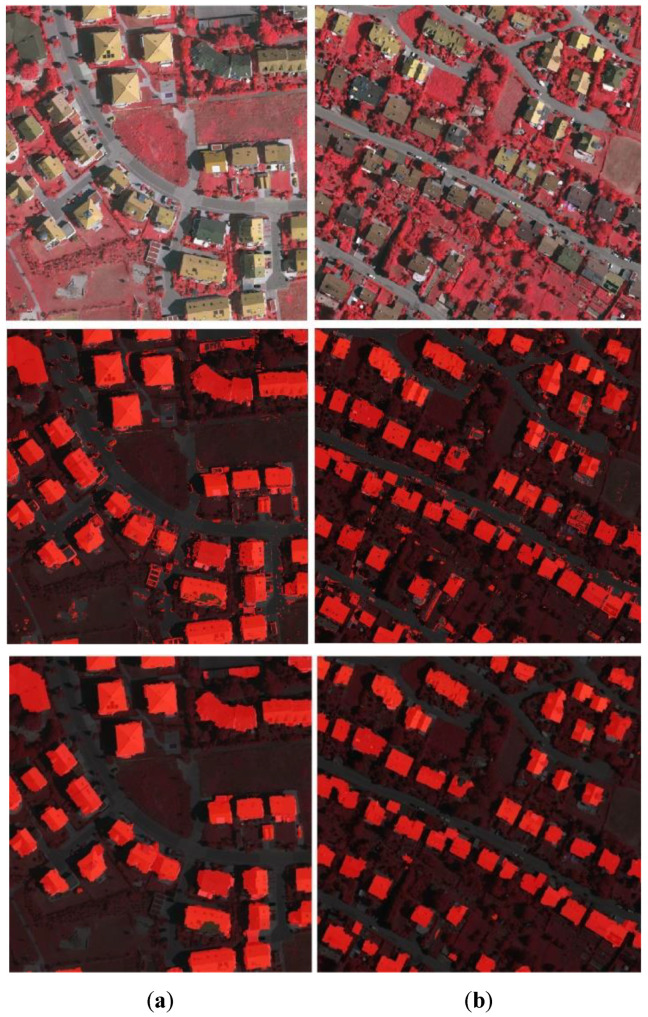
Experiment results: (**a**) results of the ISPRS simulated dataset a, (**b**) results of the ISPRS simulated dataset b (the first row is the HRS images, the second row is the initial classification results, and the third row is the final classification results).

The detection results of the ISPRS datasets are presented in Figure. 8. The middle row of Figure 8 presents the initial classification results. The bottom row of Figure 8 shows the results after optimization by using a graph cuts algorithm. Initial results show that most of the new buildings are detected. However, these building labels have holes and gaps that undermine OA. Moreover, in areas that share similar spectral textual characteristics with the buildings, such as bare soil and roads, spurious and fragmented building labels occur. This results in a high FDR. After optimization, more pure building extraction results are obtained.

#### 3.2.2. Results with Different Label Noise Levels

**Figure 9:**
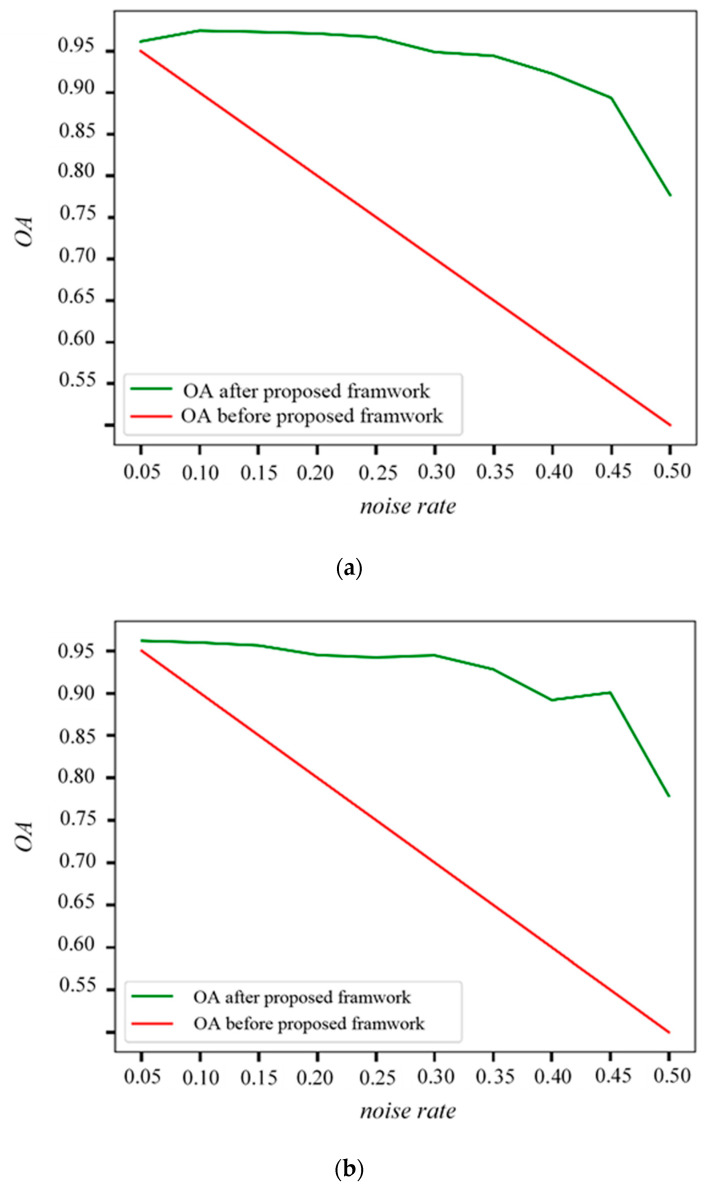
Overall accuracy w.r.t different simulated noise levels: (**a**) results of ISPRS dataset a, (**b**) results of ISPRS dataset b.

Here we analyze the performance of the proposed method on data sets with different levels of label noise and the overall accuracy w.r.t. different settings are explored. The HRS images, as shown in Figure 7a,b, are segmented into superpixels with the approximate size of the buildings. The labels of specified proportions of superpixels (ranging from 5% to 50%) are then selected randomly and flipped to introduce different levels of noise. The whole procedure of the proposed method is then performed on these modified data sets, and the results are presented in Figure 9.

The results indicate that for noise rates up to 40%, the overall accuracy of the proposed method is above 90%. Even in cases where the original noise rate reaches as high as 50% (which means the information provided by outdated basemaps are mixed), the proposed framework is able to obtain an accuracy of 75%. This indicates the effectiveness of the proposed method.

### 3.3. Results of Boston Real Dataset

#### 3.3.1. Detection Results

**Figure 10:**
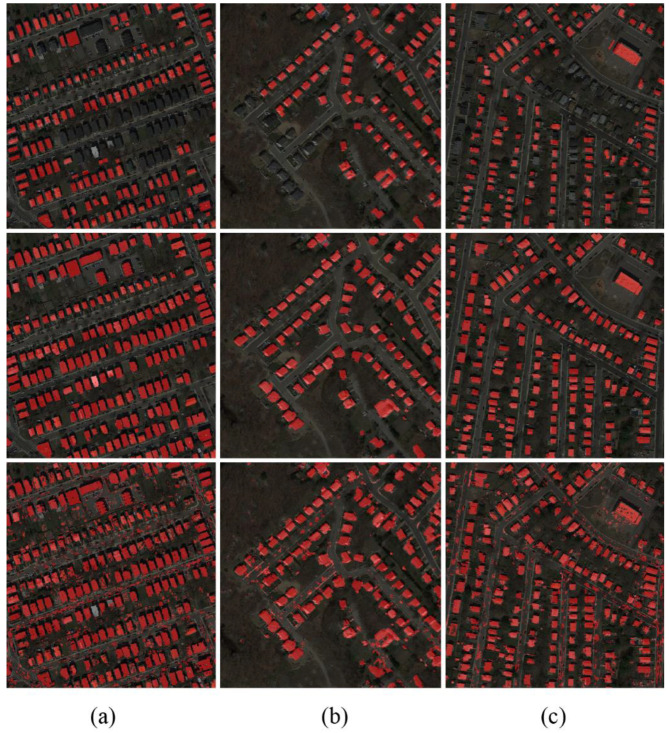
Initial classification result: (**a**) results of Boston real dataset c, (**b**) results of Boston real dataset d, (**c**) results of Boston real dataset e (first row—outdated basemap; middle row—groundtruth; third row—data cleansing result).

Figure 10 shows the outcomes of the initial classification results of Boston real datasets. Comparing the results obtained by the proposed method (the middle row of Figure 10) and the ground truth map (the bottom row of Figure 10), it is seen that most of the new buildings are correctly detected, and mis-registration errors are corrected. However, these building labels have holes and gaps that undermine OA.

**Figure 11:**
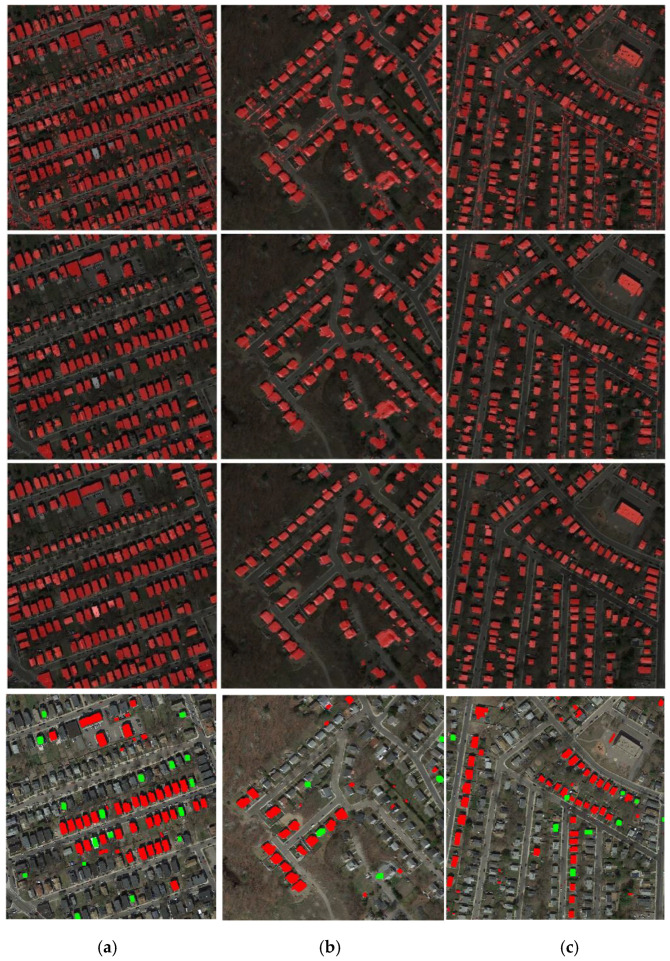
Results after post-optimization: (**a**) results of Boston real dataset c, (**b**) results of Boston real dataset d, (**c**) results of Boston real dataset e (first row—building label maps before optimization by object-based analysis and graph cuts; middle row—building label maps optimized by object-based analysis and graph cuts; third row—building map ground truth; fourth row—change map, where red means the changes are correctly detected, green means they are not).

After optimization using graph cuts, the results are presented in the third row of Figure 11. Compared with the first row in Figure 11, it is seen that the phenomenon of small segments is removed, and the building extraction results are more accurate. Based on the optimized classification results, we obtain the change maps and compare them with the ground truth of the change map. The results are shown in the fourth row of Figure 11, where the red color means the changes are correctly detected, and the green means the changes are not detected.

#### 3.3.2. Performance Comparison

In order to demonstrate the effectiveness of the proposed method, comparisons are made to three benchmarking methods, namely, A, B, and C. Method A employs the same framework as the proposed method, but uses conventional spatial-spectral features by combing GLCM textural features and normalized RGB, to replace the feature detector in our method. Method B employs a deep feature extractor as in Reference [24], and then follows the following steps: (1) Segmentation of the HRS images into superpixels; (2) cropping the bounding box of each superpixel, feeding it into ImageNet pre-trained VGGNet, extracting 4096-dimensional features from fc7, and reducing them to 100-dimensional using principal component analysis; (3) cleansing the data using graph cuts optimization. Method C is a fully pixel-based method that directly uses pixel-wise re-predicted label map for graph cuts optimization.

For the four methods to be comparable, the receptive field of features is set to 15, which is the same as the proposed method. Meanwhile, all the hyperparameters are determined through a grid search to obtain the highest performance. The accuracy results are shown in Table 2. The results confirm that the proposed method overperforms methods A, B, and C.

Compared with the proposed method, Method A shows a lower AR and a higher FDR. This shows that the deep features perform better than the hand-crafted features. Method B employs an earlier deep feature extraction strategy, however its performance on the experiment data is very low. The reason is that the buildings in the used datasets are generally small; this leads to two problems in direct segmentation of the HRS images into objects and in data cleansing: (1) The number of building samples is severely decreased, therefore, enough information is unavailable to distinguish background from the building; (2) a single building only consists of few superpixels, this makes the building objects vulnerable to the instability of random classifiers and/or over-smoothing by surrounding background objects. Nevertheless, with additional pixel-wise graph cuts post-processing in Method C, the accuracy remains low compared to the initial classification result. This is because the graph cuts algorithm punishes adjacent pixels with different labels and the correction of spurious clique needs lots of energies. Therefore, they cannot be corrected through max-flow optimization of the energy function. On the contrary, holes in building labels and fragmentations in non-building areas may dilate, leading to decreasing AR and OA.

All the experiments were performed on a laptop computer with Intel Core i7-7700HQ at a 2.8 GHz CPU with 32 GB memory, and an NVIDIA GTX1060MAXQ GPU (with 6.0 GB memory). The processing time is about five minutes for the three real data sets.

## 4. Conclusions and Future Works

In this paper, we proposed a novel framework for image-map building change detection. First, we demonstrated the representative ability of the features extracted from the early convlayer of pre-trained DCNNs and proved the feasibility of selecting important features using outdated building basemaps. Then, a random forest-based data cleansing method was implemented to preliminarily detect and correct changed pixels. The pixel-level re-predicted label maps were, however, fragmented, therefore, we adopted object-based analysis to introduce contextual information and ameliorate spurious predictions. We then used a graph cuts algorithm to optimize the label assignment results.

There are some limitations in the proposed method; for instance, a sparse distribution of the buildings may result in omission errors. Since FCFE demonstrates high efficiency in dense feature descriptors, it can be used in other tasks, such as classification and image registration [51].

## Figures and Tables

**Table 1:** Details of newly acquired HRS images in five datasets.

Dataset		Source	Size (pixels)	Spatial Resolution (m)
ISPRS simulated dataset	a	Aerial	1996 × 1995	0.09
	b	Aerial	2818 × 2558	0.09
Boston real dataset	c	Google Earth	1031 × 1097	1
	d	Google Earth	1132 × 1139	1
	e	Google Earth	1159 × 1179	1

**Table 2:** Comparison Results.

Method	Dataset (c)			Dataset (d)			Dataset (e)		
	Comp	FDR	OA	Comp	FDR	OA	Comp	FDR	OA
Proposed	**0.861**	**0.269**	**0.942**	**0.878**	**0.268**	**0.966**	**0.890**	**0.223**	**0.963**
A	0.736	0.645	0.798	0.784	0.732	0.822	0.762	0.733	0.761
B	0.419	0.495	0.874	0.246	0.594	0.919	0.304	0.600	0.887
C	0.746	0.431	0.896	0.759	0.372	0.948	0.755	0.468	0.907
